# Managing fisheries for maximum nutrient yield

**DOI:** 10.1111/faf.12649

**Published:** 2022-02-17

**Authors:** James P.W. Robinson, Kirsty L. Nash, Julia L. Blanchard, Nis S. Jacobsen, Eva Maire, Nicholas A.J. Graham, M. Aaron MacNeil, Jessica Zamborain‐Mason, Edward H. Allison, Christina C. Hicks

**Affiliations:** ^1^ Lancaster Environment Centre Lancaster University Lancaster UK; ^2^ Institute for Marine and Antarctic Studies University of Tasmania Hobart Tasmania Australia; ^3^ Centre for Marine Socioecology University of Tasmania Hobart Tasmania Australia; ^4^ Technical University of Denmark National Institute of Aquatic Resources Lyngby Denmark; ^5^ Ocean Frontier Institute Department of Biology Dalhousie University Halifax Nova Scotia Canada; ^6^ College of Science and Engineering James Cook University Townsville Queensland Australia; ^7^ ARC Centre of Excellence for Coral Reef Studies James Cook University Townsville Queensland Australia; ^8^ Harvard T.H. Chan School of Public Health Boston Massachusetts USA; ^9^ WorldFish Jalan Batu Maung Batu Maung Bayan Lepas, Penang Malaysia; ^10^ School of Marine and Environmental Affairs University of Washington Seattle Washington USA

**Keywords:** fisheries management, food security, nutrition, overfishing, seafood, sustainable fisheries

## Abstract

Wild‐caught fish are a bioavailable source of nutritious food that, if managed strategically, could enhance diet quality for billions of people. However, optimising nutrient production from the sea has not been a priority, hindering development of nutrition‐sensitive policies. With fisheries management increasingly effective at rebuilding stocks and regulating sustainable fishing, we can now begin to integrate nutritional outcomes within existing management frameworks. Here, we develop a conceptual foundation for managing fisheries for multispecies Maximum Nutrient Yield (mMNY). We empirically test our approach using size‐based models of North Sea and Baltic Sea fisheries and show that mMNY is predicted by the relative contribution of nutritious species to total catch and their vulnerability to fishing, leading to trade‐offs between catch and specific nutrients. Simulated nutrient yield curves suggest that vitamin D, which is deficient in Northern European diets, was underfished at fishing levels that returned maximum catch weights. Analysis of global catch data shows there is scope for nutrient yields from most of the world's marine fisheries to be enhanced through nutrient‐sensitive fisheries management. With nutrient composition data now widely available, we expect our mMNY framework to motivate development of nutrient‐based reference points in specific contexts, such as data‐limited fisheries. Managing for mMNY alongside policies that promote access to fish could help close nutrient gaps for coastal populations, maximising the contribution of wild‐caught fish to global food and nutrition security.

**Table:** 

1.	Introduction	801
2.	Materials and Methods	801
2.1	Multispecies size‐spectrum models	801
2.1.1	Nutrient yield curves	802
2.2	Fishing for nutrients	802
2.3	Predicting nutrient management potential	802
3.	Results & Discussion	803
3.1	multispecies Maximum Nutrient Yield	803
3.2	Nutrient yield in ecosystem models	804
3.3	Optimising catches of nutritious species	804
3.4	A nutrient vulnerability framework for catch data	805
3.5	Using mMNY to enhance production of nutritious seafood	807
3.6	Developing mMNY for data‐limited fisheries	808
3.7	Conclusion	808
	Acknowledgements	809
	Conflict of Interest	809
	Data Availability Statement	809

## 1 INTRODUCTION

Fisheries provide an essential source of dietary micronutrients (such as iron and vitamin A) and long‐chain fatty acids (such as omega‐3) to nearly 20% of the global population (Golden et al., 2016; Hicks et al., 2019; Thilsted et al., 2016). Yet micronutrient deficiencies remain prevalent globally (FAO, 2020), contributing to childhood mortality (Black et al., 2013) and early death (Afshin et al., 2019). Fisheries production policies can help close population‐level nutrient gaps if they support a sustainable increase in the production of fish rich in target nutrients (Thilsted et al., 2016), but there is currently no framework that integrates nutritional outcomes into fisheries management. Doing so requires new methods that shift the focus away from catch volumes and towards nutrient yields that meet dietary needs, helping fisheries to contribute effectively towards ending malnutrition (Bennett et al., 2021; Hall et al., 2013).

Contemporary fisheries management is founded on catch‐based reference points that quantify the maximum sustainable yield (MSY) available from single‐species stocks (Costello et al., 2016; Hilborn et al., 2020) and occasionally as the multispecies maximum sustainable yield (mMSY) (Briton et al., 2019). MSY has been pivotal in rebuilding fish catches in multiple locations (Hilborn et al., 2020) but can conflict with management objectives when species or stocks have ecological or social values that are compromised by fishing at maximum sustainable catch limits, such as conservation status or economic profitability (Andersen et al., 2015; Jacobsen et al., 2017; Matsuda & Abrams, 2006). As a result, maximum economic yield (Dichmont et al., 2010) and ecosystem indicators (Jennings, 2005; Shin et al., 2005) have been developed to understand synergies and trade‐offs between MSY and distinct management objectives. These tools are known to improve stock status (Hilborn et al., 2020) and achieve positive economic, social and environmental outcomes for fishing‐dependent communities (Asche et al., 2018). Yet, despite recent attention on the role of wild fisheries in global nutrition security (Bennett et al., 2021; Farmery et al., 2021; Golden et al., 2016; Hicks et al., 2019), the relevance of MSY for nutritional outcomes has not been explored.

Fish vary widely in their nutrient content (Tacon & Metian, 2013) according to species’ life‐history traits, phylogeny and environmental conditions (Hicks et al., 2019; Robinson et al., 2022; Vaitla et al., 2018). In a multispecies fishery, the nutrient yield of catches is likely dependent on the nutrient content of the most targeted and abundant stocks, and on gear selectivity for those stocks. Thus, depending on which mix of species are caught in what quantities, nutrient yield can be distinct from total catch weight (Hicks et al., 2019) and MSY‐based fisheries management may not optimise nutrient production. Development of management approaches that evaluate nutrient outcomes is essential if wild capture fisheries are to contribute meaningfully to securing global food and nutrition security (Farmery et al., 2021).

Here, we develop a nutrient‐sensitive approach to management of wild capture fisheries. We develop a conceptual framework to estimate multispecies Maximum Nutrient Yield (mMNY) for fisheries and examine potential trade‐offs with mMSY. Next, we combine nutrient content data (Hicks et al., 2019) with two empirically validated multispecies models that have been used to explore fisheries policy objectives for the North Sea (Blanchard et al., 2014) and Baltic Sea (Jacobsen et al., 2017). Following previous use of these models, our analysis is designed for strategic use in evaluating management approaches, here to demonstrate a proof‐of‐concept for mMNY, rather than tactical use in setting catch limits and evaluating uncertainty in parameter estimation (Blanchard et al., 2014; Plagányi et al., 2014). We generate nutrient yield curves and compare mMNY, mMSY and ecosystem status across different exploitation levels. Based on the nutrient curves and species composition in North and Baltic Sea models, we then propose ways to identify fisheries that offer greatest potential for optimising nutrient production and apply this approach to catch data for global marine fisheries.

## 2 MATERIALS AND METHODS

### 2.1 Multispecies size‐spectrum models

Size‐based models were used to examine nutrient production by multispecies fisheries, construct nutrient yield curves and determine nutrient reference points. We fit a dynamic multispecies size‐spectrum model to simulate a generic assemblage of 15 interacting species with varying nutrient concentrations, a North Sea fish assemblage of 12 interacting pelagic and demersal species (Blanchard et al., 2014) and a Baltic Sea fish assemblage of 3 interacting pelagic and demersal species (Jacobsen et al., 2017). Species are defined by species‐specific life‐history parameters, and allometric scaling rules are used to scale individual processes (growth and mortality) to population‐ and community‐level size structure (Andersen et al., 2016; Jacobsen et al., 2014). Energy flux is accounted for between all individual processes for a full energy budget. Fishing mortality is imposed by combining size‐selectivity curves with historic estimates of fishing mortality. The North Sea model additionally uses species co‐occurrence data to model competitive interactions (Blanchard et al., 2014). We used models calibrated to historic catch and biomass data (Figures S1, S2), with parameterisations described in full in (Blanchard et al., 2014; Jacobsen et al., 2017). All simulations were implemented in R (R Core Team, 2021), using the *mizer* package for the North Sea (Table S1) (Scott et al., 2014).

#### 2.1.1 Nutrient yield curves

We generated yield curves by simulating a range of exploitation rates from unfished to collapse of community biomass (10% of unfished community biomass) or of most stocks (>50% of stocks collapsed). In mizer models, fishing mortality (*F*) is the product of selectivity (*S*), catchability (*Q*) and relative fishing effort (*E*),

$$F_{g,i}\left(w\right)=S_{g,i}\left(w\right)Q_{g,i}E_{g}$$

for each gear (*g*) and species (*i*) at size class *w*, per year. *S* is defined by a trawl size‐selectivity function applied to each species across gear types (North Sea: industrial, pelagic, beam, otter; Baltic Sea: small, medium, large). By setting *Q* at 1, we used the fishing effort parameter *E* to consistently change fishing mortality across gears while holding their relative species and size selectivities constant (Scott et al., 2014). Using models calibrated to historic fishing intensity (Figs. S1, S2) and run to equilibrium, we simulated a range of fishing mortalities, relative to each species simulated F at MSY (F_MSY_ year^−1^). For North Sea, simulated F_MSY_ values were identified by increasing fishing effort on each species while holding other species to their estimated F_MSY_ in ICES stock assessments (Thorpe et al., 2015). For Baltic Sea, simulated F_MSY_ values were identified by fitting a state‐space Pella‐Tomlinson model to observed catch and biomass. We then generated yield curves using an effort multiplier, whereby we fished each species at its simulated F_MSY_ value multiplied by *E* and increased *E* from 0 (unfished) to fishing levels that depleted community biomass (<10% unfished biomass) or more than 50% of stocks. At each value of *E*, we estimated exploitation rate at the community level (total catch/total biomass) and extracted catch by species (tonnes per year) and fish community indicators (mean size, community fish biomass). Nutrient yields were each species’ total catch multiplied by its nutrient concentration. We focus on four minerals (calcium, iron, selenium and zinc), vitamins A and D and omega‐3 fatty acid levels that are bioavailable and important in human diets (Hicks et al., 2019). Nutrient concentrations were estimated for each species using a trait‐based nutrient model (minerals, omega‐3 and vitamin A) (Hicks et al., 2019) and from food composition tables (vitamin D) (Norwegian Food Safety Authority, 2020; Public Health England, n.d.; Schmid & Walther, 2013).

Nutrient yields were summed across species to generate nutrient yield curves across the range of fishing mortality. As in (Worm et al., 2009), we estimated community fish biomass, mean maximum size (cm) and the number of stock collapses, defined as species that fell below 10% of their unfished biomass. From these curves, we identified the multispecies Maximum Sustainable Yield (mMSY, maximum total catch) and multispecies Maximum Nutrient Yield (mMNY, maximum catch of each nutrient), and the community‐level fishing mortality required to produce these values (F_mMSY_ and F_mMNY_ respectively). Catch, nutrient and ecosystem reference points were visualised by rescaling yield curves, biomass and mean size as a proportion of their maximum.

### 2.2 Fishing for nutrients

Nutrient yield curves were generated by setting relative fishing mortality for each species (i.e. effort multiplier relative to estimated F_MSY_). To investigate the potential for species‐specific fishing regulations to raise mMNY, we used an optimisation function to identify the fishing mortality that produced the maximum total nutrient catch, without inducing stock collapses. We used an optimiser to find the set of fishing mortalities (*F*) that maximised total nutrient yield, but discarded any mortality sets that caused one or more stock collapses (<10% species’ unfished biomass). Because species mortalities can vary independently of other stocks, this process can find higher catch than predicted by nutrient yield curves, provided those mortalities do not induce stock collapse. *F* was bound between 0.01 and 2 and optimised using *optim* in R, with a quasi‐Newton algorithm. Models were optimised separately for each nutrient in each model (i.e. 7 nutrients, each for North Sea and Baltic Sea models). We visualised the effect of these fishing strategies by examining the change in the nutrient yield, total catch and fishable biomass relative to their average (simulated) values between 2000 and 2010. We also measured the change in *F* on each species relative to its estimated averaged fishing mortality over 2000–2010, thus indicating which species were fished more or less to achieve mMNY in the optimisation process.

### 2.3 Predicting nutrient management potential

**FIGURE 1:**
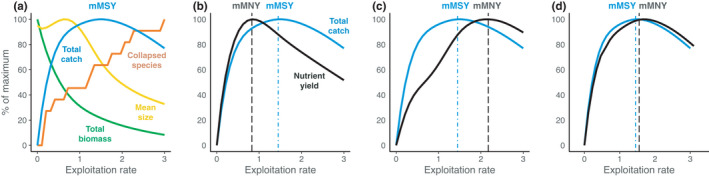
Theorised maximum nutrient yield curves for multispecies fisheries. (a) shows the effect of exploitation rate on total catch (blue), fishable biomass (green), mean size (yellow) and number of collapsed stocks (orange). Nutrient yield curves may be maximised at fishing levels (b) below mMSY (nutrient overfishing), (c) above mMSY (nutrient underfishing) or (d) similar to mMSY. Catch curves were generated using a generic size‐based fisheries model of 15 interacting species with varying nutrient concentrations

Our model simulations revealed that the distribution of nutrient catches among species (i.e. evenness) and species’ vulnerability to fishing influenced the relationship between mMSY and mMNY. We therefore define an evenness ~vulnerability framework that produces three management scenarios—(1) mMSY is approximately equal to mMNY, (2) mMSY is above mMNY (nutrient overfishing) and (3) mMSY is below mMNY (nutrient underfishing), as conceptualised in Figure 1. We test this framework with data by estimating (1) the evenness of nutrient catches among species and (2) the mean fishing vulnerability of species, weighted by each species’ nutrient catch. First, we validated this framework using simulated North Sea and Baltic Sea mMSY and mMNY reference points. We estimated the evenness and vulnerability of simulated catch at each nutrient's mMNY, and for total catch at mMSY. Catch evenness was Pielou's evenness (Shannon diversity/log(species richness)), and catch vulnerability was the mean of all single‐species F_MSY_, weighted by each species’ total catch or nutrient catch. Nutrients were resilient (i.e. nutrient underfishing), if the nutrient‐weighted mean F_MSY_ was above the total catch F_mMSY_, and vulnerable (nutrient overfishing), if the nutrient‐weighted mean F_MSY_ was below the total catch F_mMSY_.

We then applied this framework to the Sea Around Us (SAU) database of marine fisheries catches (Pauly et al., 2020) to identify the vulnerability of nutrient catches to fishing, following (Maire et al., 2021). These data include fisheries of varying exploitation status, including both managed and unregulated stocks. As such, we use our evenness‐vulnerability framework to identify potential trade‐offs between catch and nutrients, based on current catch levels, rather than to quantify nutrient‐based catch limits. We extracted all reconstructed fish catches from Exclusive Economic Zones (EEZ) of 185 countries, excluding discards and averaged over 2010–2014, for species‐, family‐ and genera‐level records. Following (Hicks et al., 2019), we estimated the concentration of six nutrients (four minerals, omega‐3 and vitamin A) for each species in the SAU database, using traits from Fishbase (Froese & Pauly, 2020) (vitamin D estimates were not available for most marine fish in SAU), and quantified the yield of each nutrient for each catch record. Catches without species‐level information were assigned the mean nutrient value of species in the lowest level taxonomic group (family or genus), and fishing vulnerability was defined using an index of species’ intrinsic vulnerability to fishing (Cheung et al., 2005). For each country, we then estimated (1) the evenness of each nutrient catch and (2) the fishing vulnerability of each nutrient catch, weighted by species (Maire et al., 2021). Using our evenness‐vulnerability framework, we assessed the potential for multispecies (high nutrient‐catch evenness) and single‐species approaches to nutrient‐based management (low nutrient‐catch evenness) and identified countries with nutrient catches that were resilient or vulnerable to fishing. We also examined species composition in the 20 most uneven nutrient catches, as we expected these to be most suitable for nutrient‐based management of relatively few stocks. Finally, we examined the potential for nutrient under‐ and overfishing by measuring the fishing vulnerability of each nutrient catch relative to total catch, for each country. This metric indicated if nutrients were supplied by species that were more or less vulnerable to fishing than those that dominated total catch, thereby revealing potential trade‐offs between catch and nutrients within a country's EEZ.

## 3 RESULTS & DISCUSSION

### 3.1 multispecies maximum nutrient yield

In a multispecies fishery, annual catches can increase with exploitation rate up to a maximum total catch (i.e. mMSY, at fishing mortality F_mMSY_), leading to reductions in average body size, depleted community biomass and an increased risk of stock collapses (Figure 1A). Catches of nutrients also increase with exploitation rate, up to a maximum nutrient catch, enabling the estimation of multispecies maximum nutrient yield (mMNY, at F_mMNY_) that depends on the nutrient content of landed species. We outline three potential outcomes of fishing for mMSY versus mMNY. First, nutrient yields may be maximised below F_mMSY_ when nutrient‐rich species are vulnerable to overfishing, such that reducing total catch is required to produce mMNY (Figure 1B), representing nutrient overfishing (F_mMNY_ < F_mMSY_). Second, maximum nutrient yields above F_mMSY_ may arise when nutrient‐rich species are resilient to high exploitation rates, producing the largest nutrient yield at fishing levels that cause stocks of less nutrient‐rich species to collapse (Figure 1C), representing nutrient underfishing (F_mMNY_ > F_mMSY_). Finally, catches of nutrient‐rich species may be maximised at mMSY, such that nutrient yields correlate closely with total catches (Figure 1D; F_mMNY_ ≈ F_mMSY_). Application of this conceptual mMNY model requires understanding the nutrient composition of a multispecies fishery, with mMNY curves varying among locations and nutrients according to the traits of target species and their relative abundance and vulnerability to fishing.

### 3.2 Nutrient yield in ecosystem models

Size‐based models of North Sea and Baltic Sea fisheries were used to construct nutrient yield curves for seven nutrients (calcium, iron, selenium, zinc, omega‐3 fatty acids and vitamins A and D). These nutrients are important for human health and bioavailable in fish, three of which are often lacking in diets in European countries (selenium, omega‐3 and vitamin D) (Cashman et al., 2016; Stark et al., 2016; Stoffaneller & Morse, 2015). We use these fisheries systems because they have been assessed and modelled in various contexts (Blanchard et al., 2014; Jacobsen et al., 2017; May et al., 1979; Ulrich et al., 2016) and because these stocks are already targeted, traded and consumed, providing a firm foundation to evaluate nutrient‐based management in a multispecies context. Both models simulate fishing on species that grow and interact according to simple size‐based metabolic scaling rules, calibrated to historic exploitation rates, and have provided strategic advice on long‐term management objectives (Blanchard et al., 2014; Jacobsen et al., 2017). North Sea fisheries were modelled with four gear types exploiting 12 pelagic and demersal species, with lesser sandeel (*Ammodytes marinus*, Ammodytidae), herring (*Clupea harengus*, Clupeidae) and plaice (*Pleuronectes platessa*, Pleuronectidae) the largest contributors to total catches across a range of fishing mortality. North Sea mMSY was reached when sandeel was fished below its single‐species MSY, and plaice and herring were fished close to single‐species MSY (Figure S3). Baltic Sea fisheries were modelled with trawl gears exploiting three species, with herring and sprat (*Sprattus sprattus*, Clupeidae*)* contributing most to total catch and mMSY reached at fishing mortality above sprat F_MSY_ and below herring F_MSY_ (Figure S3).

**FIGURE 2:**
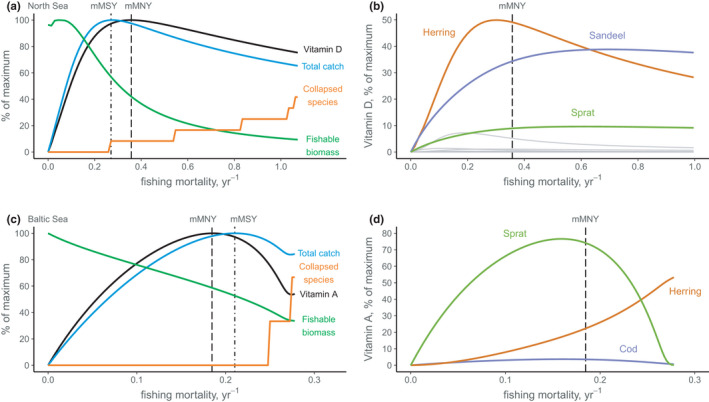
Nutrient yield curves in North Sea and Baltic Sea fisheries. (a) In the North Sea, maximum vitamin D yield occurred at fishing mortality above F_mMSY_, owing to (b) high contribution of sandeel (purple), herring (orange) and sprat (green) to vitamin D yields. The remaining nine species (grey) contributed <5% of the maximum vitamin D yield owing to their low productivity and/or low vitamin D concentration. (c) In the Baltic Sea, maximum vitamin A yield occurred at fishing mortality below F_mMSY_, owing to relatively high contribution of (d) sprat and herring to selenium yields. Fishing mortality is total catch/total biomass

We found potential for nutrient underfishing in both fisheries models. In the North Sea, vitamin D yields were maximised at exploitation levels above mMSY (Figure 2A). Almost 80% of the maximum vitamin D yield was provided by two species (sandeel and herring), with sandeel particularly resilient to moderate fishing mortality (Figures 2B, S3). With total catch maximised at relatively light fishing mortality but higher vitamin D concentrations in resilient sandeel and herring (Figure S4), vitamin D yield reached mMNY at fishing mortality 40% above F_mMSY_ (Figure 2B). In the Baltic Sea, iron and selenium yield were maximised at fishing levels 8% and 11% above F_mMSY_, respectively, and four nutrients were maximised at fishing levels that collapsed sprat and cod stocks (Figure 2C, S5). High fishing mortality maximised yields of these nutrients following overfishing or collapse of cod and sprat stocks (Figure S6), inducing predation release and high productivity for herring, which were concentrated in multiple nutrients (Figure S7). In contrast, nutrient overfishing was only detected in two nutrients. Selenium was maximised at 90% of F_mMSY_ in the North Sea, with maximum yield reached when cod, sandeel and herring contributed to catch, before cod collapse (Figure S6). In the Baltic Sea, vitamin A was maximised below mMSY (88% of F_mMSY_) (Figure S5), owing to most nutrient yields being provided by one species (sprat) (Figure S6) which reached its maximum catch at fishing mortality below F_mMSY_ (Figure S3). mMSY fishing levels may therefore underfish most nutrients in North Sea and Baltic Sea fisheries, and overfish selenium (North Sea) and vitamin A (Baltic Sea). In all simulations, fishing above F_mMSY_ and F_mMNY_ triggered stock collapses, causing substantial reductions in both total catch and nutrient yields.

For most nutrients, yield at mMSY was within 93% of MNY, indicating that fishing close to mMSY would achieve a ‘pretty good’ multispecies nutrient yield. In systems where nutrient curves closely follow catch curves, fishing levels that return a pretty good yield could therefore provide an operating space for maximising both catch and nutrients (Hilborn, 2010; Rindorf et al., 2016). Alternatively, catch and nutrient curves might also diverge, causing larger differences between catch and nutrient yield. For example, underfishing North Sea stocks returned 67% of maximum vitamin D yield at 78% of mMSY, whereas in the Baltic Sea, overfishing levels returned 54% of maximum vitamin A yield at 84% of mMSY (Figure 2A, C). Yield curves can therefore be used to predict the magnitude of differences between catch and nutrients and thus to assess scenarios that might provide most nutrients at low fishing levels, or when overfishing might cause a disproportionate loss of nutrient yield.

### 3.3 Optimising catches of nutritious species

Uptake of mMSY approaches in fisheries management has been limited by both model complexity and practical barriers to implementation, such as interactions between gears and species, social and economic drivers, and historical exploitation patterns (Thorpe et al., 2016). However, in a multispecies fishery with stocks of varying nutrient concentrations, mMNY is governed by the sustainable yield of nutritious species only and therefore may be simpler to implement than mMSY in some systems. For example, yield of vitamin D (North Sea) and vitamin A (Baltic Sea) was highly dependent on sandeel and sprat catches respectively. Optimising yields of single species that return the highest nutrient production might therefore be the most effective method of maximising nutrient production in some multispecies contexts. The potential benefits to public health through mMNY will likely also depend on consumer preference and availability of seafood products, as, for example, sandeel and sprat are currently not directly consumed in Europe. Alternatively, nutrient yield curves may require management of multiple nutritious species to raise mMNY. Selenium, for instance, was dependent on catches of several stocks in both fisheries models and, in the Baltic Sea, zinc was maximised when cod and sprat were removed from the system, releasing herring from predation and competition (Andersen et al., 2015). We found that optimising fishing mortality to maximise nutrient yields from stocks that contributed most to nutrient yield curves raised nutrient catch by 28%–156% above historic catch levels (average simulated 2000–2010), without collapsing any stocks (Figure S8). In these simulations, mMNY was achieved by allowing fishing mortality to vary independently of species’ F_MSY_, which indicated that increasing fishing on the most nutritious and productive species, such as Norway pout, herring and sandeel, could enhance nutrient yield (Figure S8). Regulating fishing for nutrient catch also raised total catch by 63% (34%–93%) but decreased fishable biomass by 40% (30%–50%) (mean, minimum and maximum across nutrients and models) and was achieved by overfishing predators and exploiting subsequent high productivity of forage fish. Our optimised catches exceeded historic levels because the single‐species MSY approaches used in North Sea and Baltic Sea fisheries protect yields of individual stocks and therefore avoid such fishing‐induced trophic cascades.

Maximising nutrients, therefore, presents a potentially important trade‐off for fisheries management, alongside catch, economic and environmental outcomes, and thus an additional dimension to integrate in fisheries management. As with trade‐offs between catch and nutrients, biodiversity impacts of fishing for nutrients will depend on the resilience of nutritious species to fishing. For example, cases of nutrient underfishing in North Sea and Baltic Sea models were usually associated with stock collapses and substantially depleted fishable biomass at mMNY (Figure S5), suggesting these scenarios would also impact ecosystem structure and biodiversity. In a mMNY assessment, such impacts could be assessed with ecosystem indicators (e.g. large fish indicator, biodiversity and food web stability) (Briton et al., 2019). Nevertheless, fishing regulations that promote catches of nutritious and productive species could sustainably raise specific nutrient yields and therefore should be considered as a management strategy that prioritises nutrition security over ecosystem or economic objectives in certain nutrient‐deficient scenarios. The size‐based models used here therefore provide strategic advice on long‐term policy objectives for fisheries management, rather than to identify nutrient‐based reference points for North Sea and Baltic Sea fisheries. As with all reference points, developing mMNY for policy decisions would require these trade‐offs to be quantified and communicated, for example by evaluating uncertainty in nutrient concentrations and catch estimators (Thorpe et al., 2016) and incorporating interactions between metiérs (e.g. gear, fleet, fishing zone and season) (Hoshino et al., 2018). Tactical advice of nutrient‐based reference points for these fisheries would, for example, require models with spatial structure (e.g. species distributions and fishing grounds) and the ability to evaluate technical interactions between fisheries (Ulrich et al., 2016).

### 3.4 A nutrient vulnerability framework for catch data

Our North and Baltic Sea simulations revealed that the relative ability of nutritious species to withstand fishing pressure is a key determinant of mMNY curves. Nutrient‐based management is therefore most relevant to fisheries where caught species vary with respect to nutrient composition and vulnerability to fishing. This also implies that systems with similar species but distinct productivity levels, species interactions and fishing metiers have different catch compositions and therefore system‐specific mMNY curves. For example, three species (cod, herring and sprat) influenced nutrient yields in both North Sea and Baltic Sea models but, as community composition and fishing effects varied between regions, these systems had distinct sets of mMNY curves.

**FIGURE 3:**
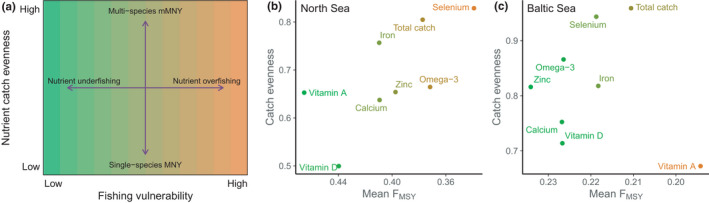
Predicting Maximum Nutrient Yield from catch evenness and species’ vulnerability to fishing. (a) In fisheries with high nutrient‐catch evenness, nutrients are supplied by several species, such that reaching mMNY will require fishing effort to be optimised over multiple species. In fisheries with low nutrient‐catch evenness, few or one species contribute to nutrient yields, such that single‐species management might be used to achieve MNY. In both multispecies and single‐species contexts, nutrient catches that are dominated by species resilient to fishing will have F_mMNY_ > F_mMSY_, such that nutrients are underfished at mMSY. Nutrient catches that are dominated by species vulnerable to fishing will have F_mMNY_ < F_mMSY_, indicating nutrients are overfished at mMSY. (b) North Sea vitamin D yield was more uneven and less vulnerable to fishing than total catch, indicating nutrient underfishing at F_mMSY_ where few species contributed to nutrient yields. (c) Baltic Sea vitamin A yield was more vulnerable to fishing than total catch at mMSY, indicating nutrient overfishing at F_mMSY_ where few species contributed to nutrient yields. Points are the catch evenness and mean F_MSY_ for each nutrient and total catch at mMSY, coloured by F_MSY_ (orange = vulnerable, green = resilient). F_MSY_ scales (b, c) are reversed to correspond with fishing vulnerability in (a) (i.e. high F_MSY_ = low fishing vulnerability)

We use these observations to develop a framework for assessing the potential for nutrient overfishing or underfishing in catch data (e.g. Figure 1), where the potential for nutrient yields to deviate from mMSY is jointly predicted by the distribution of nutrient catches among species and the mean single‐species F_MSY_ for each nutrient catch. A nutrient is expected to be overfished when those species have low F_MSY_ (i.e. relatively high fishing vulnerability) and underfished when the most nutritious species have high F_MSY_ (i.e. relatively low fishing vulnerability) (Figure 3A). In addition, fisheries with a nutrient catch that is more evenly distributed among species are likely to be characterised by multispecies nutrient yield curves, whereas uneven distributions of nutrient catches suggest single‐species MNY approaches would be most effective. We applied this nutrient vulnerability framework to the North Sea and Baltic Sea models by quantifying the evenness (Pielou's index) and vulnerability to fishing (defined as each species’ simulated F_MSY_) of nutrient catches at mMNY and total catch at mMSY. As predicted, nutrients that maximised near to mMSY in simulations also had vulnerability to fishing similar to total catch, such as North Sea calcium and zinc (Figure 3B) and Baltic Sea iron (Figure 3C). Nutrients identified as nutrient overfishing in our simulations (Figure S5) had more uneven nutrient catch and higher vulnerability to fishing than total catch (selenium in the North Sea; vitamin A in the Baltic Sea) (Figure 3B, C), whereas cases of nutrient underfishing had mean single‐species F_MSY_ above total catch F_MSY_ (North Sea vitamins A and D, Baltic Sea selenium, omega‐3 and zinc) (Figure 3B, C). Our framework therefore predicted cases of nutrient over‐ and underfishing that were broadly consistent with mMNY of simulated catch curves (Figure S9), simply using information on each species’ (simulated) F_MSY_ and nutrient catch at F_mMSY_.

**FIGURE 4:**
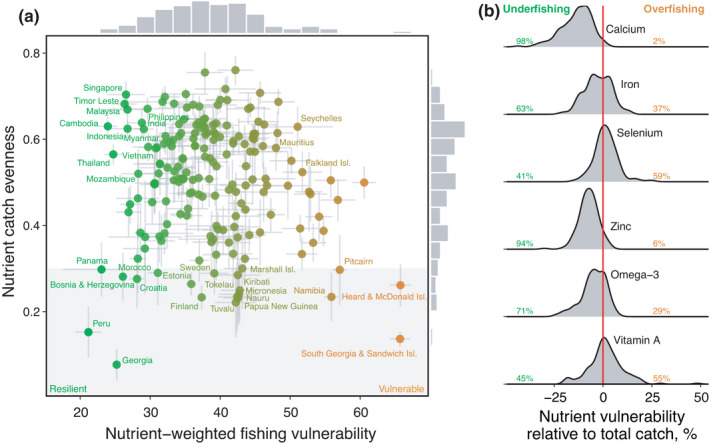
Nutrient‐catch evenness and vulnerability of commercial marine catches from EEZs of 185 countries. (a) Points are the mean evenness and vulnerability to fishing of nutrient catches across six nutrients (calcium, iron, selenium, zinc, omega‐3 fatty acids and vitamin A) (±2 SEM), coloured according to their vulnerability to fishing from resilient (green) to vulnerable (orange). Labelled points indicate countries with even catches that were particularly resilient (<30) or vulnerable (>50), as well as the 20 most uneven countries (shaded area). Marginal histograms show data distributions along each axis. (b) Density plots show the vulnerability to fishing of nutrient catch relative to total catch, for each nutrient among all 185 countries. Distribution shading and annotated percentages indicate the proportion of countries where species that provided nutrient catch are less (negative values) or more (positive values) vulnerable to fishing than species that provided total catch, indicating potential nutrient under‐ or overfishing respectively

To understand the potential global scope of our evenness‐vulnerability framework, we used the Sea Around Us Project (SAUP) data set of reconstructed commercial marine fisheries catches (Pauly et al., 2020) to identify regions where nutrient yields were skewed towards vulnerable or resilient species, as defined by a standardised fishing vulnerability metric (Cheung et al., 2005). This global catch analysis is used to identify EEZs where nutrient yields are provided by few or multiple species and whether those species are vulnerable or resilient to fishing, irrespective of differences in fishing pressure, fleet composition, gear type and management intensity among stocks. For catches in Exclusive Economic Zones (EEZ) of 185 countries, we quantified the mean nutrient‐catch evenness and intrinsic fishing vulnerability (index from 0 to 100) (Cheung et al., 2005) of commercial marine catches of six nutrients, for which species‐level concentration data were available (calcium, iron, selenium, zinc, omega‐3 fatty acids and vitamin A) (Hicks et al., 2019). Dietary intake of these nutrients is estimated to be inadequate globally (Afshin et al., 2019; Beal et al., 2017), yet all six nutrients are concentrated and bioavailable in fish (Hicks et al., 2019). Our framework predicts that countries with high catch evenness will likely require multispecies approaches to maximise nutrient yield and, using landings data, this suggests that mMNY may be most effective in locations where nutrient catches were particularly resilient (e.g. South East Asia) or vulnerable to fishing (e.g. western Indian Ocean) (Figure 4A).

In contrast, countries with uneven catches depended upon relatively few stocks for nutrient yields, such that single‐species management approaches might be the most effective method of achieving mMNY. In 20 countries with the most skewed nutrient‐catch distributions, regions with high catches of tuna species (tribe *Thunnini*, Scombridae; Pitcairn), horse mackerel (*Trachurus capensis*, Carangidae; Namibia) or Patagonian toothfish (*Dissostichus eleginoides*, Nototheniidae; Antarctic Ocean territories) had very high vulnerability to fishing (Figure S10). Nutrient catch was resilient to fishing in only six countries where catches were dominated by anchovy (Engraulidae sp.), sardine (*Sardina pilchardus*, Clupeidae) and tropical herring (*Opisthonema libertate*, Clupeidae) (Figure S11), whereas Baltic Sea and tropical Pacific countries were dominated by (moderately) resilient species, such as sprat and skipjack tuna (*Katsuwonus pelamis*, Scombridae) respectively (Figure S12). Thus, catch reconstructions suggest that fisheries development of relatively few stocks in these EEZs could further enhance nutrient yields by prioritising maximum sustainable catch of either vulnerable (e.g. large tuna species) or resilient (e.g. forage fish) stocks. Dominance of resilient species in catches may also reflect historic overexploitation (Cheung et al., 2007), such as in the Baltic Sea, where stocks of nutritious species may already be depleted. Catch time‐series data could be used to identify locations where nutrient productivity has already been compromised by overfishing (e.g. recovering, overfished and collapsed stocks), enabling managers to enhance nutrient production by prioritising recovery of those stocks.

Having identified how evenness and vulnerability interact, we next examined the vulnerability of nutrient catch relative to total catch within each country to understand if current catch levels are likely to lead to nutrient under‐ or overfishing. Globally, calcium and zinc yields were less vulnerable than total catch in 98% and 94% of countries (Figure 4B), respectively, indicating that these nutrients are concentrated in productive, resilient species that are under‐represented in current catch. Managing multispecies fisheries for single‐species MSY, used for ~98% of species in SAUP (Skern‐Mauritzen et al., 2016), might therefore result in lost catch potential of calcium and zinc, for which inadequate dietary intakes are prevalent across Asia, the Pacific and sub‐Saharan Africa, particularly for women (Balk et al., 2017; Beal et al., 2017). Other nutrients deviated less consistently from total catch, with iron, selenium, omega‐3 and vitamin A catches indicating potential for both nutrient under‐ and overfishing. Our analysis thus indicates that mMNY‐based management could enhance nutrient yields in many of the world's fisheries.

### 3.5 Using mMNY to enhance production of nutritious seafood

While there is broadscale recognition of the need to transition towards healthy and sustainable diets (Willett et al., 2019) through a comprehensive food systems approach (Ruel, Alderman, & Maternal and Child Nutrition Study Group, 2013; Ruel et al., 2018), current policy focusses primarily on consumers (Afshin et al., 2019). Conversely, development of models and approaches to estimate mMNY in specific contexts would support enhanced production of essential dietary nutrients, particularly in places where the fishery composition leads to differences between mMSY (or multiple single‐species MSY plans) and mMNY for one or more micronutrients that are deficient in diets. For example, the North Sea and Baltic Sea vitamin D catch curves are particularly relevant in Europe, where over 10 million people are vitamin D deficient (Cashman et al., 2016). The high nutrient productivity of just two North Sea species indicates that optimising catches for vitamin D and developing policies that support the inclusion of herring and sandeel into local diets could have significant public health benefits. Similarly, regulating cod stocks for selenium production could help promote consumption of locally caught selenium‐rich seafood in European countries with suboptimal selenium intakes (Stoffaneller & Morse, 2015).

While mMNY for Northern European fisheries is most relevant for selenium and vitamin D, undernutrition in many locations is caused by inadequacies in multiple micronutrients (Beal et al., 2017). In these contexts, mMNY can help address hidden hunger by combining nutrient yield curves, for example, to assess fishing levels that maximise specific vitamins, minerals or all nutrients combined (Figure S13). mMNY would therefore help fisheries managers to prioritise overall nutrient production, complementing efforts to enhance food and nutrition security through greater access to fish (Thilsted et al., 2016). In the tropical Pacific, for instance, policies that allocate abundant pelagic species for local consumption have been proposed to support a growing population while relieving pressure on climate‐impacted coastal fisheries (Bell et al., 2018). These policies could be coupled with nutrient outcomes by focussing management attention on the most nutrient‐rich pelagic species. Fisheries managed for nutrient outcomes will therefore require support from markets and institutions to raise demand for nutritious seafood and promote access to fish. Indeed, nutrition‐sensitive agriculture programmes that link crop production to markets, education and health have delivered positive nutrition outcomes (Ruel et al., 2018), suggesting that demand and consumption of nutritious seafood can be shaped by nutrition‐sensitive fisheries policies.

The effectiveness of mMNY‐based management will also depend on the influence of international trade and foreign fishing, which drive extensive global movement of seafood from point of capture (Watson et al., 2017) and thus shape nutrient supply. These distribution processes may exacerbate nutrition insecurity by exporting fishery‐derived nutrients from nations suffering nutrient deficiencies. For example, foreign fleets catch and export large volumes of nutritious pelagic fishes from West African EEZs (Belhabib et al., 2015), removing nutritious seafood from places where inadequate intakes are most prevalent (Hicks et al., 2019) and where fisheries may already be vulnerable to nutrient overfishing (e.g. Namibia, Figure S10). Seafood trade networks also direct large quantities of forage fish towards aquaculture feed rather than direct human consumption, such as in Peru, where almost 90% of catch is Peruvian anchoveta (*Engraulis ringens*, Engraulidae) (Figure S11) that are mostly caught for fishmeal and fish oil (Cashion et al., 2017). Reducing wild fish into farmed products is an inefficient way of consuming fish‐derived nutrients (Willer et al., 2022) and can move nutritious fish away from nutritionally vulnerable countries to those that are nutritionally secure (Golden et al., 2017). Information on mMNY could help source nations think strategically about the nutritional consequences of fishing access agreements and account for the impact of trade agreements on local nutrient supplies (Hicks et al., 2019). Such information is critical if nations are to address nutrient deficiencies and minimise negative outcomes of foreign fishing and trade.

### 3.6 Developing mMNY for data‐limited fisheries

Optimising fisheries for nutrients will have the greatest impact in regions where wild‐caught fish are critical sources of essential dietary micronutrients, such as tropical countries with diverse small‐scale fisheries (Bennett et al., 2021). Multispecies and ecosystem models have not yet been developed for the majority of these data‐limited fisheries, restricting our ability to construct MSY curves. However, concern over the status of such unassessed fisheries, comprising >80% of global catch (Costello et al., 2012), has motivated development of reliable data‐limited stock assessment tools, most requiring only catch data and simple life‐history information to estimate MSY (Froese et al., 2017; Martell & Froese, 2013; Zhou et al., 2018). These tools could be further developed for tactical use, estimating mMNY‐based reference points using nutrient composition data from FishBase. As in our global catch analysis, information on catch composition can be converted into nutrient yields to identify the gears, fleets, and species that supply nutrient‐rich seafood and consequently help to identify fisheries that might provide a source of nutrients lacking in local diets. Indeed, most small‐scale fisheries catches are consumed locally (Kelleher et al., 2012), indicating that combining mMNY‐based management with policies that support access to nutrient‐rich fish could have a considerable impact on diet quality in places where undernutrition is prevalent.

### 3.7 Conclusion

Fisheries managed for nutrient production should aim to promote biomass of productive and nutritious stocks, provided those populations are limited by fishing (e.g. nutrient under‐ or overfishing, Figure 1). By using strategic fisheries models to develop a conceptual mMNY framework, our study is intended to motivate development of nutrient‐based fisheries reference points and the methodological tools to estimate them, particularly in data‐limited tropical systems where nutrient deficiencies are prevalent. Nutrient composition data are now available for over 6,000 fish species (github.com/mamacneil/NutrientFishbase). Combined with catches, these data can identify stocks that contribute most to nutrient yields and are available to consumers with suboptimal dietary intakes of key nutrients. These fisheries could be governed for maximum nutrient production as a global public good. Capture fisheries have reached their ecological limits for food production by volume (Costello et al., 2020) but, deployed strategically, mMNY‐based management could supply more nutritious seafood. Nutrition‐sensitive agriculture (Ruel et al., 2013; 2018) that incorporates fisheries‐derived nutrients, particularly in locations where people have access to fish but inadequate nutrient intakes, could help realise the ocean's potential as a nutritious and sustainable food system.

## CONFLICT OF INTEREST

Authors declare that they have no competing interests.

## Supporting information

Supplementary Material

## Data Availability

All data and code used in the analysis are available at github.com/jpwrobinson/MaxNutrientYield.
