# Supplementary material for: Managing fisheries for maximum nutrient yield
Source: Fish Fish (Oxf). 2022 Feb 17;23(4):800–11. doi: 10.1111/faf.12649 (PMC9303942; doi:10.1111/faf.12649)
Supplement: Supplementary file 1 — Supplementary Material [file FAF-23-800-s001.pdf]

Supplementary Figures

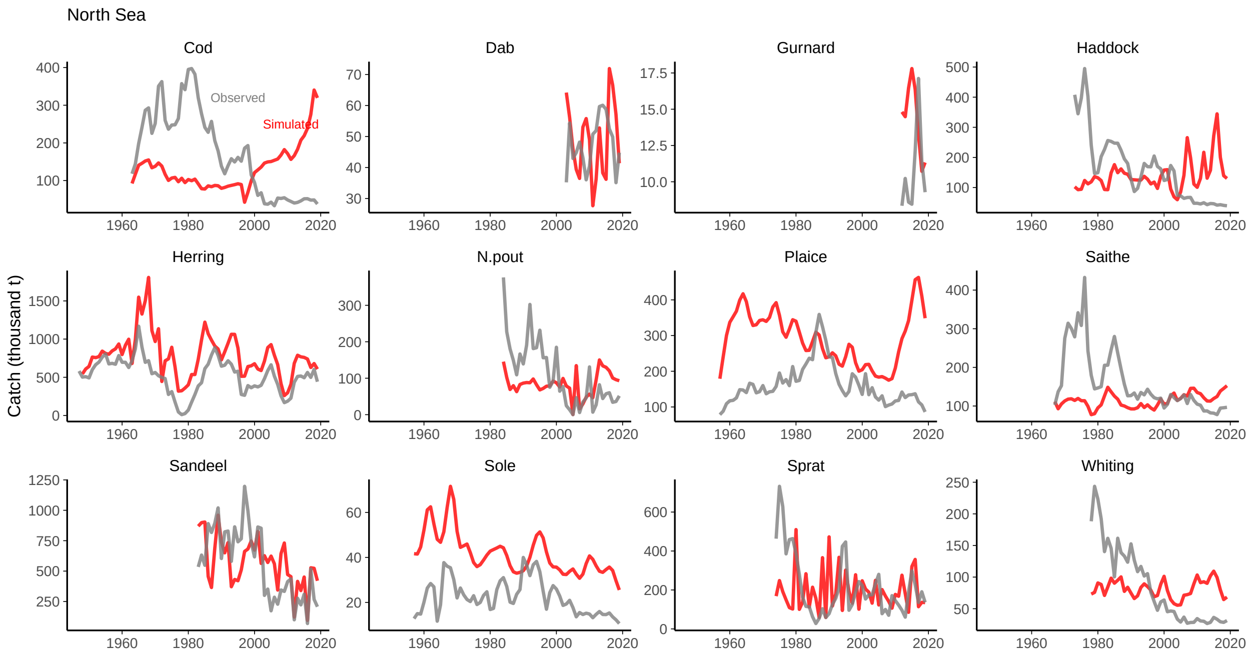

Fig. S1. North Sea model fit to historic fishing data. The North Sea model calibrated to long-term fishing mortality estimates (grey lines) reproduced patterns in catch weights from 1947 to 2019 (red lines). Model calibration described in detail by Blanchard et al. 2014 (ref. 23).

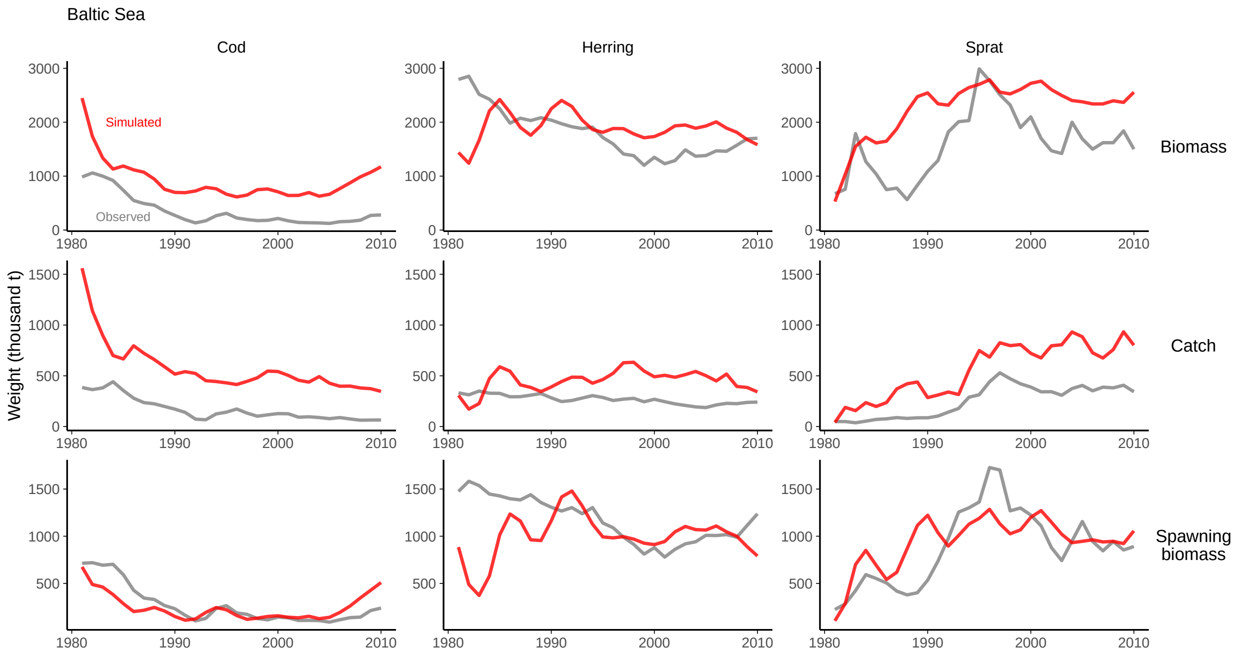

Fig. S2. Baltic Sea model fit to historic fishing data. The Baltic Sea model calibrated to long-term fishing mortality estimates (grey lines) reproduced patterns in biomass, catch weights and spawning biomass from 1981-2010 (red lines). Model calibration described in detail by Jacobsen et al. 2017 (ref. 14).

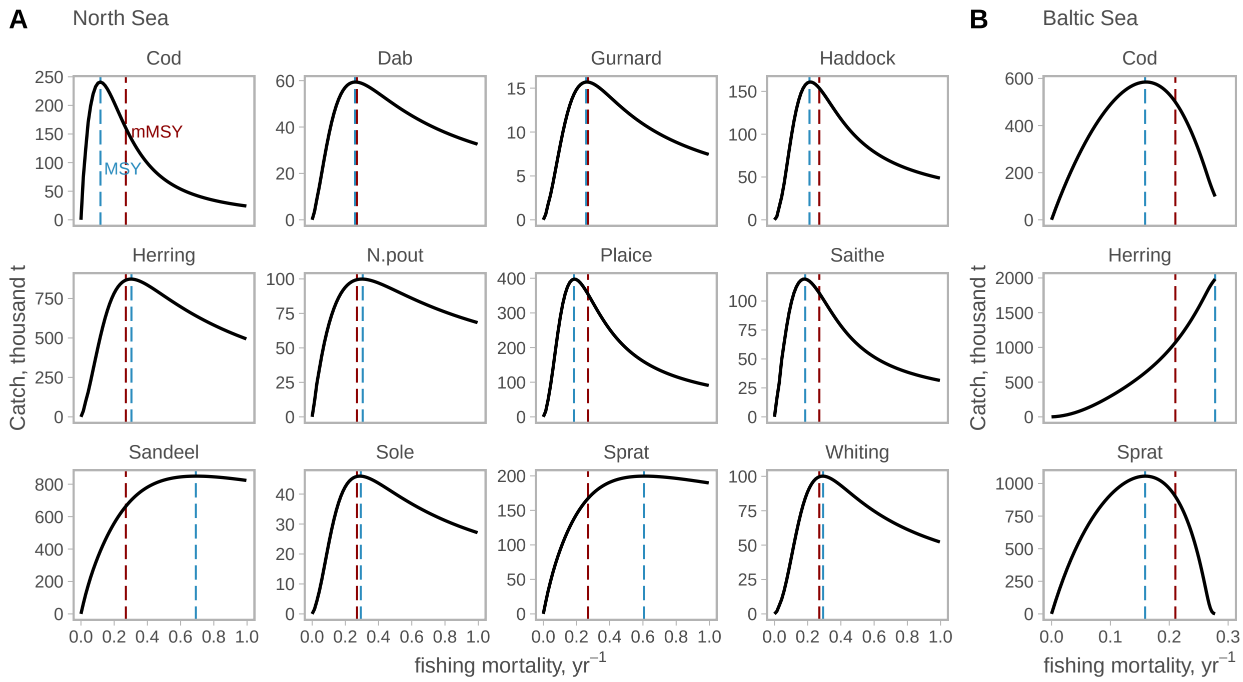

Fig. S3 Single-species catch yield curves (i.e. single-species MSY). Catch yield curves for each species in the North Sea (**A**) and Baltic Sea models (**B**). Blue dashed line is single-species F_{MSY}, the fishing mortality where each species' catch, and thus its nutrient yield, is maximised. Red dashed line is F_{mMSY}, the fishing mortality where multispecies catch is maximised (Fig. 2).

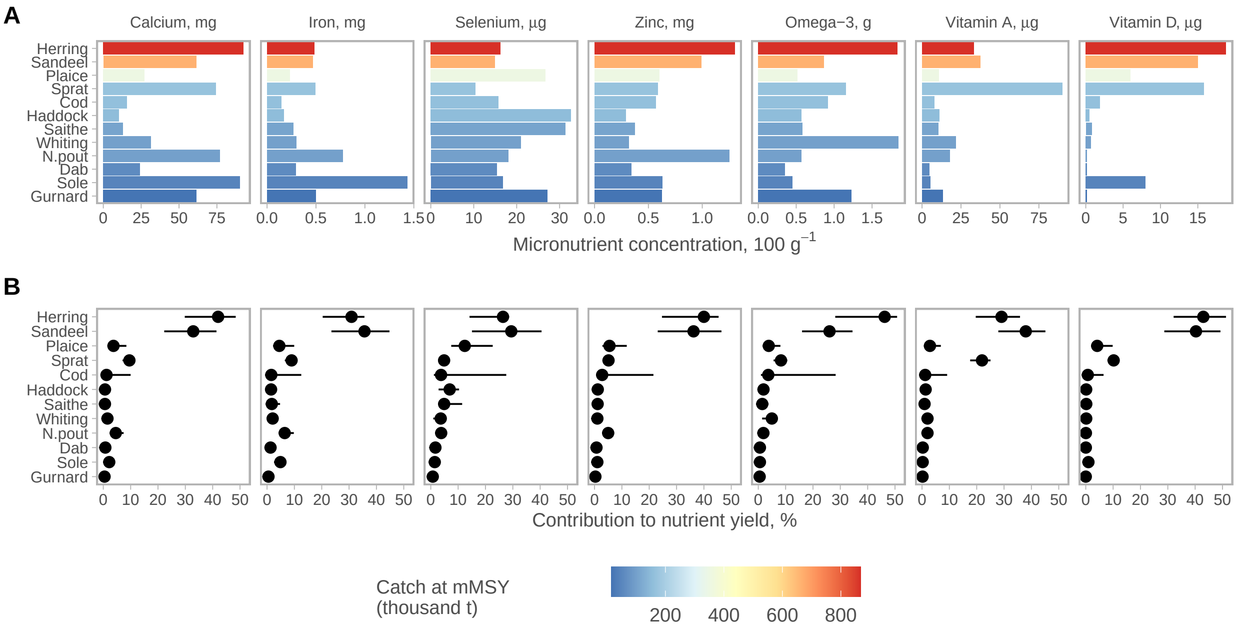

Fig. S4. Nutrient yield composition of North Sea fisheries. In (**A**) bars show concentrations of seven nutrients for 12 species in the North Sea model, ordered and coloured by each species' total catch (in thousand tonnes per year) at mMSY. In (**B**) points show the mean contribution of each species to total nutrient yields across the full nutrient yield curve (i.e. the range of simulated fishing mortality) (± minimum and maximum contributions).

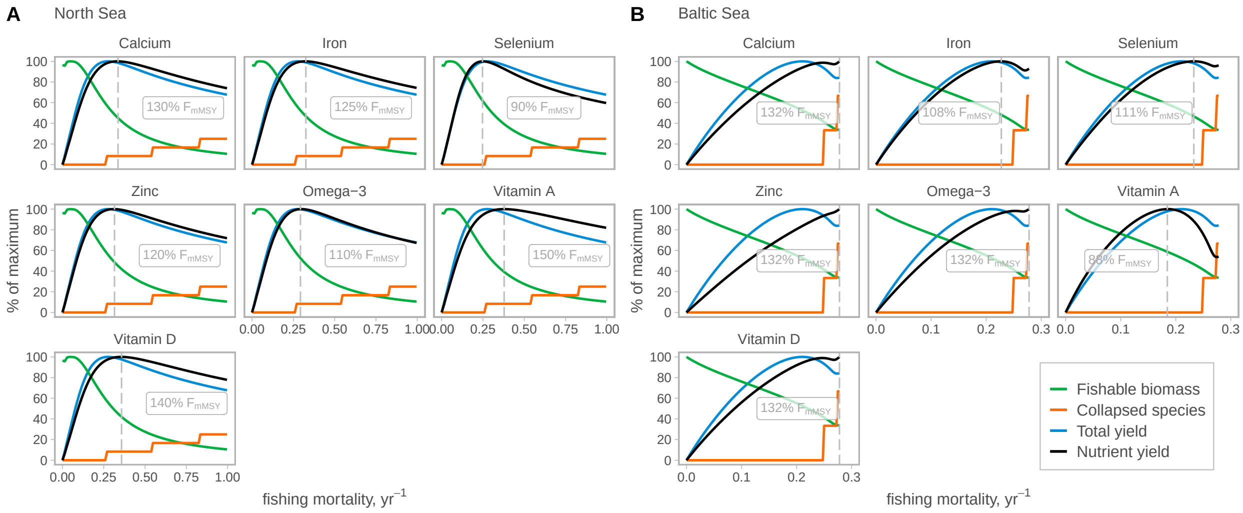

Fig. S5. Nutrient yield curves for six nutrients in the North Sea (A) and Baltic Sea (B) models. For each micronutrient (panels, black lines), nutrient yield curves are shown with the total yield (blue), fishable biomass (green) and number of collapsed species (orange). Dashed grey line is F_{mMNY} for each nutrient.

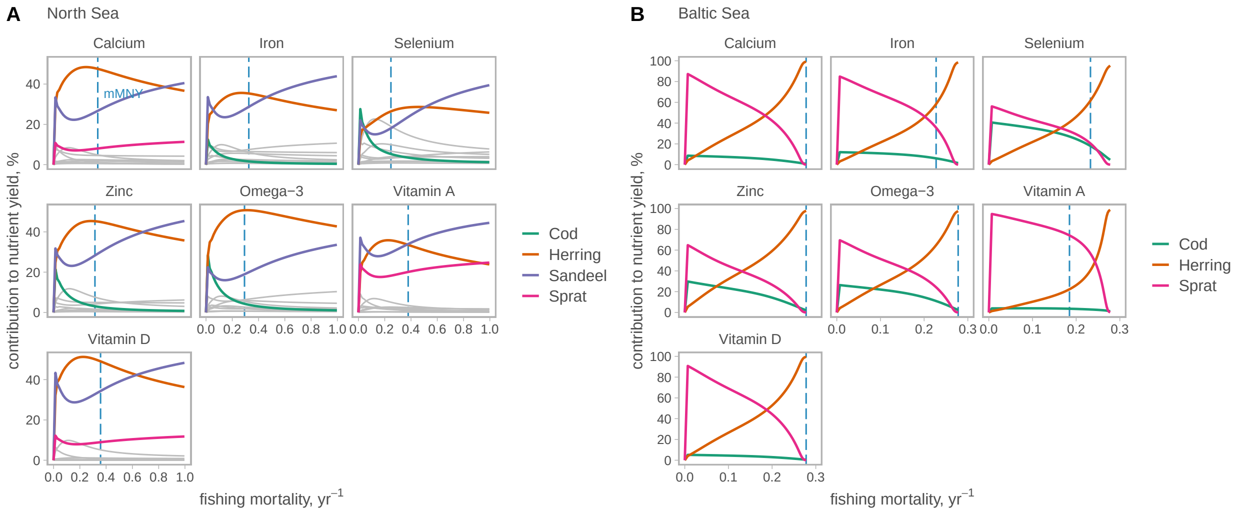

Fig. S6. Species contributing most to nutrient yields in the North Sea (A) and Baltic Sea (B). In the North Sea, coloured species are the top-3 contributors to yields of each nutrient, other species coloured grey. In the Baltic Sea, all three species are shown. mMNY for each nutrient is annotated with blue dashed lines.

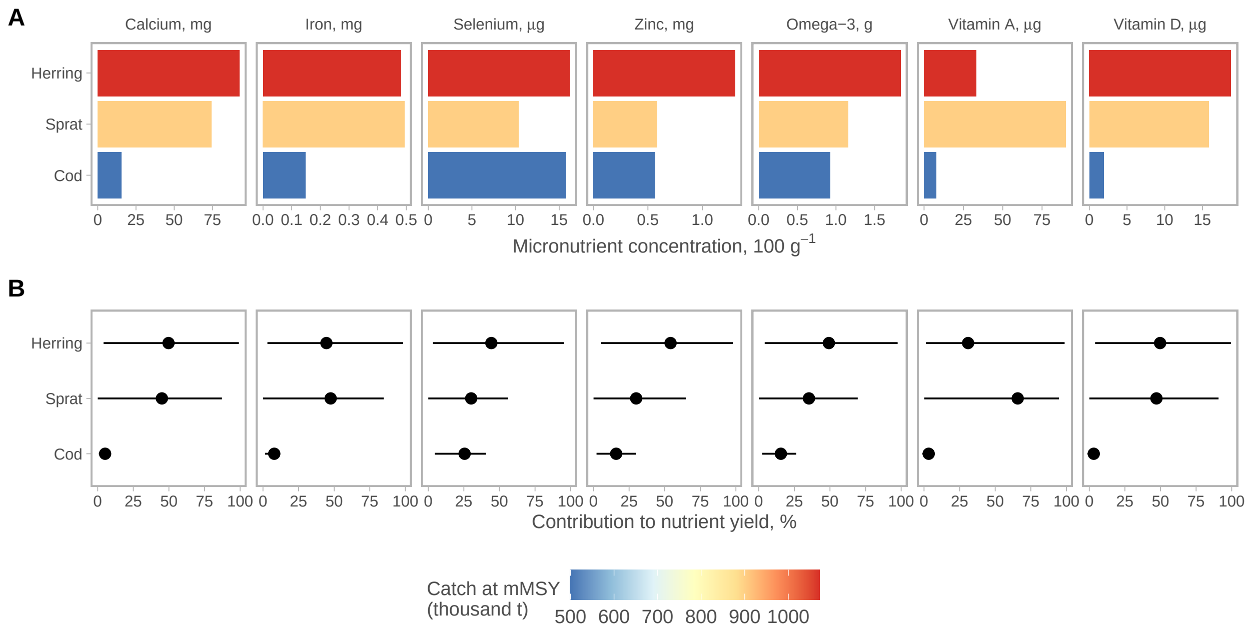

Fig. S7. Nutrient yield composition of Baltic Sea fisheries. In (**A**) bars show concentrations of seven nutrients for 3 species in the Baltic Sea model, ordered and coloured by each species' total catch (in thousand tonnes per year) at mMSY. In (**B**) points show the mean contribution of each species to total nutrient yields across the full nutrient yield curve (i.e. the range of simulated fishing mortality) (± minimum and maximum contributions).

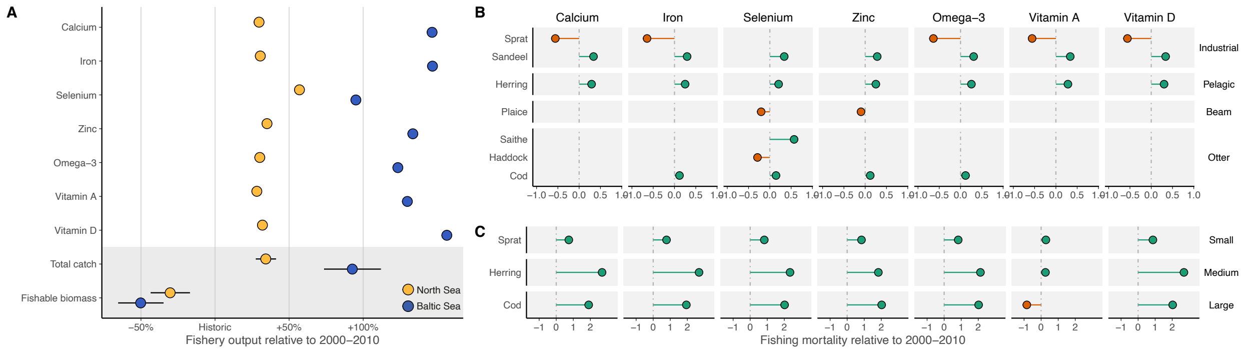

Fig. S8. Optimising fishing effort for Maximum Nutrient Yield. (A) Optimising fishing mortality to achieve mMNY could raise nutrient yield and total catch above historic levels (mean simulated 2000-2010) in both North Sea (yellow) and Baltic Sea (blue) models. Points are the nutrient yield from fisheries models optimised to maximise each specific nutrient, with total catch and fishable biomass represented by the mean fishery output among all nutrients (± 2 S.E.M.). (B,C) mMNY was achieved by optimising fishing mortality among nutritious and productive species, either increasing (green) or decreasing (orange) fishing mortality relative to historic levels (mean estimated 2000-2010). Species are ordered according to their simulated gear type, for North Sea (B) and Baltic Sea (C).

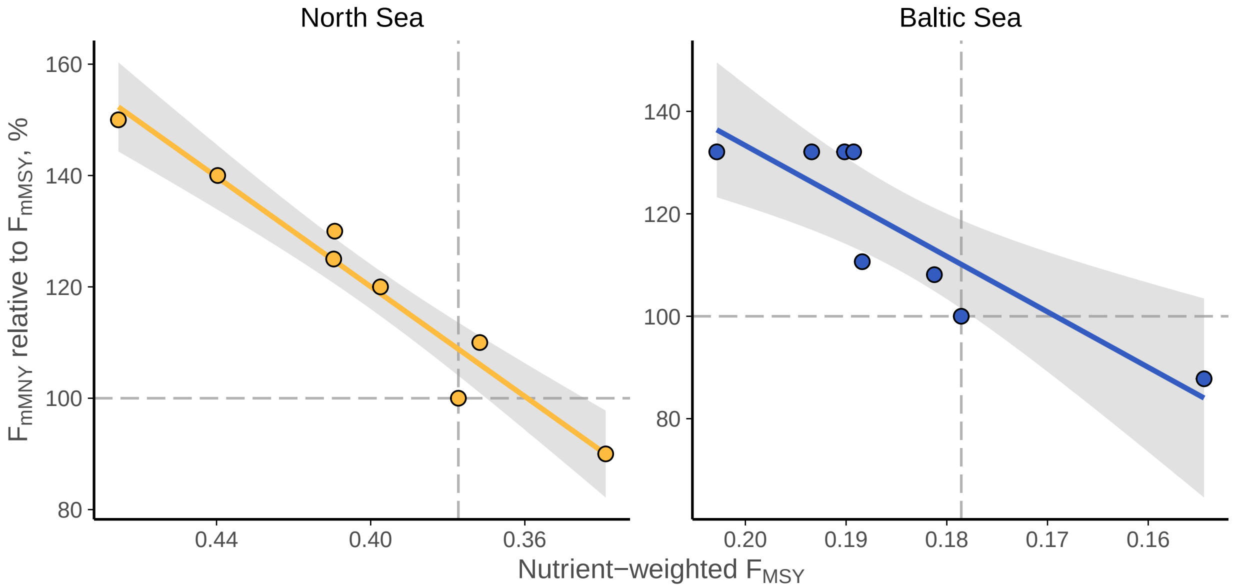

Fig. S9. Predictive accuracy of evenness-vulnerability framework. At mMSY, the vulnerability to fishing of each nutrient predicted cases of nutrient overfishing ($F_{mMNY} < F_{mMSY}$) and underfishing ($F_{mMNY} > F_{mMSY}$) in both the North Sea (yellow) and Baltic Sea (blue) models. Y-axis values are simulated F_{mMNY} relative to F_{mMSY} (e.g. Fig. S4) and x-axis values are catch-based metrics of the nutrient-weighted mean single-species F_{MSY} (e.g. Fig 3). Grey dashed lines indicate F_{mMSY} from catch curves (100% on the y-axis) and the simulated catch composition (catch-weighted mean single-species F_{MSY} on the x-axis).

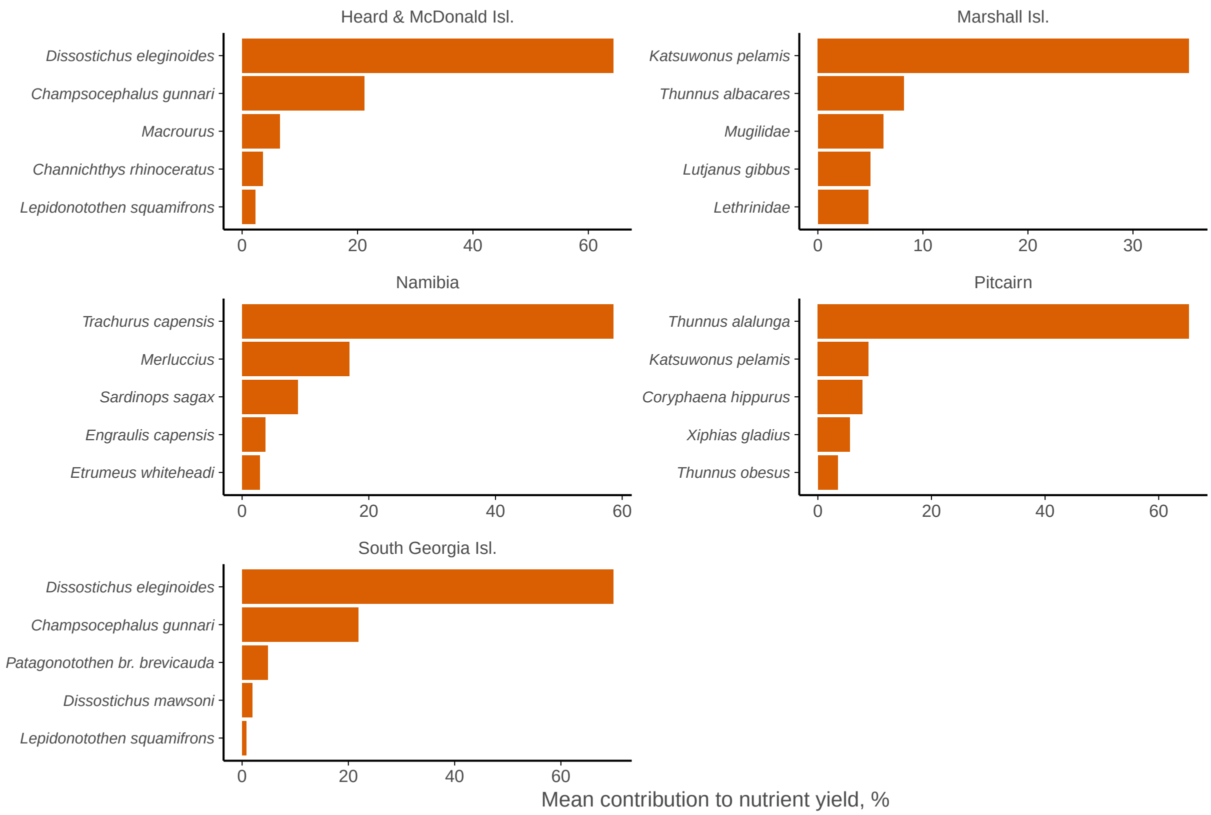

Fig. S10. Nutrient catch distribution for countries with high nutrient catch unevenness and high vulnerability to fishing. Bars are the proportion of total nutrient catch provided by the top 5 most landed species, for countries with uneven catches and a nutrient-weighted species' vulnerability to fishing above 43 (Fig. 4).

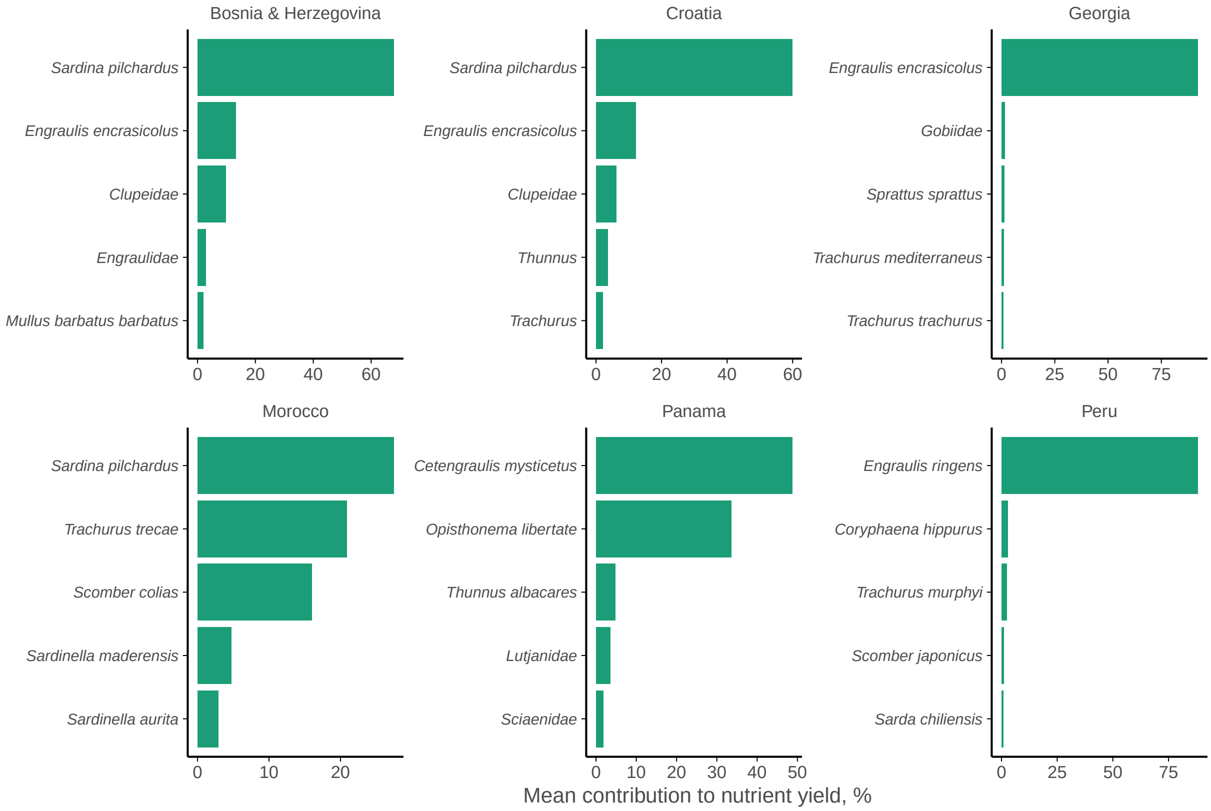

Fig. S11. Nutrient catch distribution for countries with high nutrient catch unevenness and low vulnerability to fishing. Bars are the proportion of total nutrient catch provided by the top 5 most landed species, for countries with uneven catches and a nutrient-weighted species' vulnerability to fishing below 33 (Fig. 4).

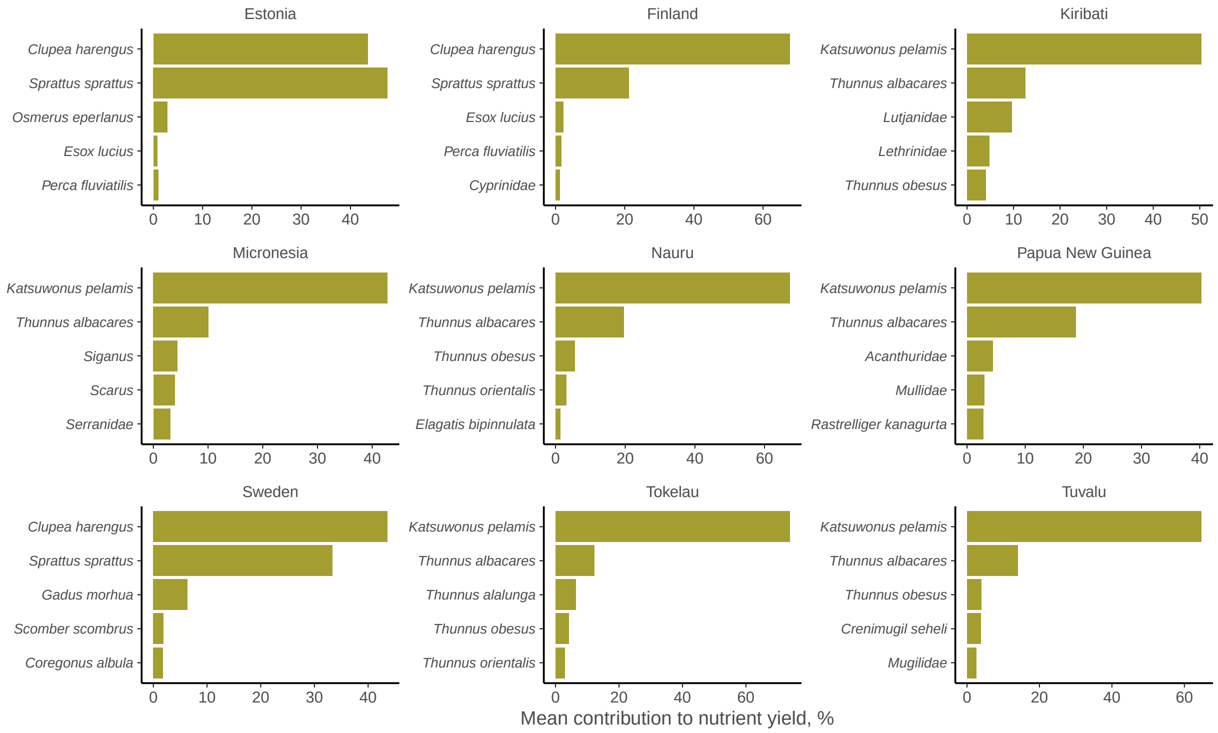

Fig. S12. Nutrient catch distribution for countries with high nutrient catch unevenness and moderate vulnerability to fishing. Bars are the proportion of total nutrient catch provided by the top 5 most landed species, for countries with uneven catches and a nutrient-weighted species' vulnerability to fishing between 33-43 (Fig. 4).

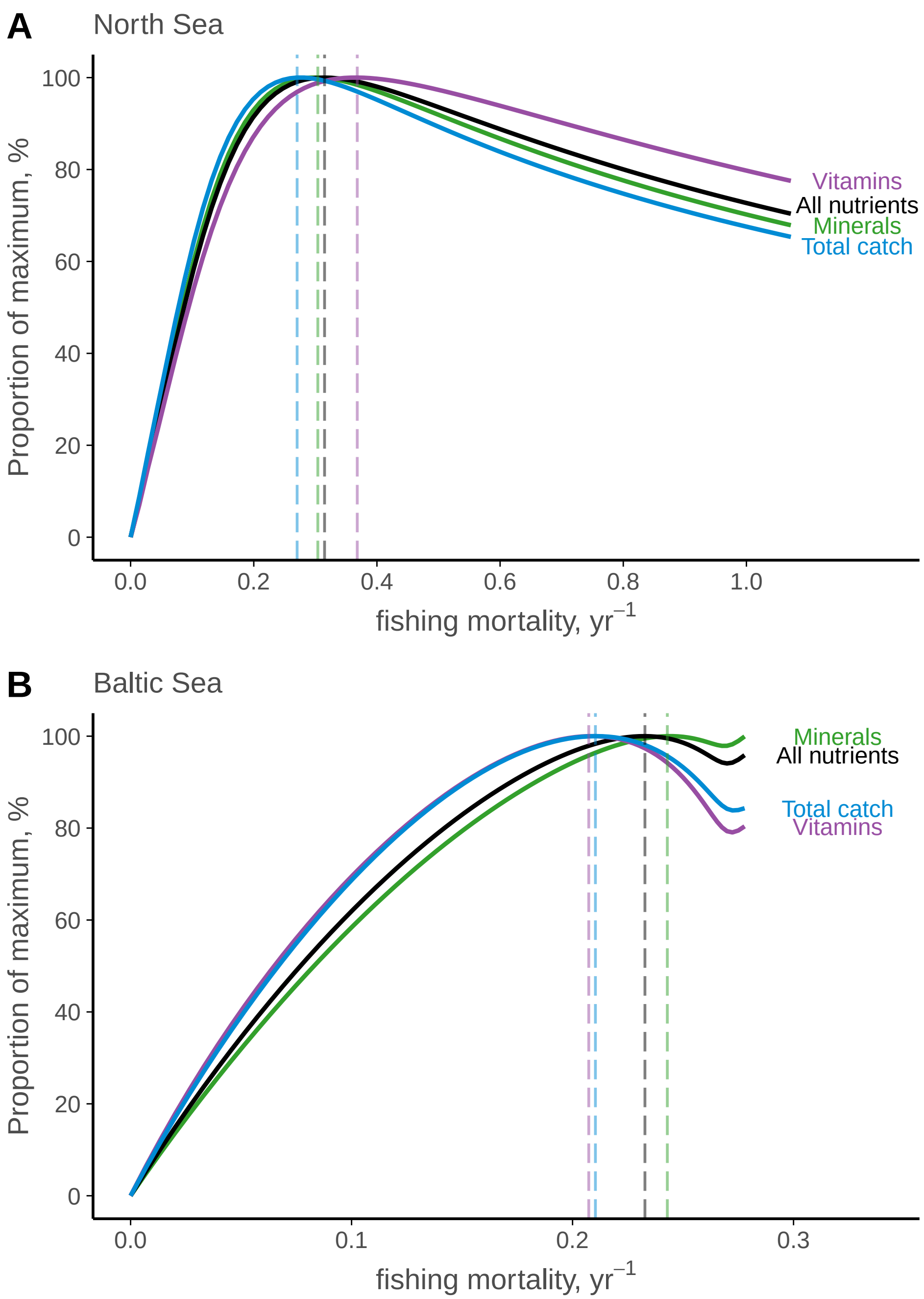

Fig. S13. Combined nutrient yield curves for North Sea (A) and Baltic Sea fisheries (B). Lines show the combined nutrient yield for vitamins (purple), minerals (green), and all nutrients (black), and total catch (blue). Dashed lines show mMNY for each combined nutrient curve.

species	w_inf	w_mat	beta	sigma	k_vb	gear	h	ks	z0	gamma	w_mat25	R_max	knife_edge_size
Sprat	33	13	51076	0.8	0.681	Industrial	18.20	3.64	0.19	3.20E-11	11.65	7.38E+11	13
Sandeel	36	4	398849	1.9	1	Industrial	27.52	5.50	0.18	1.51E-11	3.58	4.10E+11	4
N. pout	100	23	22	1.5	0.849	Industrial	32.84	6.57	0.13	8.63E-11	20.61	1.05E+13	23
Herring	334	99	280540	3.2	0.606	Pelagic	35.04	7.01	0.09	1.13E-11	88.70	1.11E+12	99
Dab	324	21	191	1.9	0.536	Beam	30.68	6.14	0.09	4.67E-11	18.82	1.12E+10	21
Whiting	1192	75	22	1.5	0.323	Otter	28.54	5.71	0.06	7.50E-11	67.20	5.48E+11	75
Sole	866	78	381	1.9	0.284	Beam	22.56	4.51	0.06	3.13E-11	69.88	3.87E+10	78
Gurnard	668	39	283	1.8	0.266	Otter	19.38	3.88	0.07	2.96E-11	34.94	1.65E+12	39
Plaice	2976	105	113	1.6	0.122	Beam	14.62	2.92	0.04	2.86E-11	94.08	4.08E+14	105
Haddock	4316.5	165	558	2.1	0.271	Otter	36.77	7.35	0.04	4.35E-11	147.83	1.84E+12	165
Cod	39851.3	1606	66	1.3	0.216	Otter	61.48	12.30	0.02	1.60E-10	1438.91	8.26E+09	1606
Saithe	39658.6	1076	40	1.1	0.175	Otter	49.73	9.95	0.02	1.64E-10	964.05	1.12E+11	1076

Table S1. Mizer parametrisation for North Sea multispecies size spectrum model. Full details in Scott et al. (2014, *Methods Ecol Evo*). Parameters not shown had a single value: w_min (0.001), alpha (0.6), pred_kernel_type (lognormal), k (0), m (1), erepro (1), sel_func (knife_edge), catchability (1), p (0.7), q (0.8), n (0.667), f0 (0.6), and interaction_resource (1).
